# Erythropoietin enhances hippocampal long-term potentiation and memory

**DOI:** 10.1186/1741-7007-6-37

**Published:** 2008-09-09

**Authors:** Bartosz Adamcio, Derya Sargin, Alicja Stradomska, Lucian Medrihan, Christoph Gertler, Fabian Theis, Mingyue Zhang, Michael Müller, Imam Hassouna, Kathrin Hannke, Swetlana Sperling, Konstantin Radyushkin, Ahmed El-Kordi, Lizzy Schulze, Anja Ronnenberg, Fred Wolf, Nils Brose, Jeong-Seop Rhee, Weiqi Zhang, Hannelore Ehrenreich

**Affiliations:** 1 Division of Clinical Neuroscience, Max Planck Institute of Experimental Medicine, Göttingen, Germany; 2 Dept. of Neurophysiology, Georg-August-University, Göttingen, Germany; 3 DFG Research Center for Molecular Physiology of the Brain (CMPB), Göttingen, Germany; 4 Dept. of Molecular Neurobiology, Max Planck Institute of Experimental Medicine, Göttingen, Germany; 5 Dept. of Nonlinear Dynamics, Max Planck Institute of Dynamics and Self-Organization, Göttingen, Germany

## Abstract

**Background:**

Erythropoietin (EPO) improves cognition of human subjects in the clinical setting by as yet unknown mechanisms. We developed a mouse model of robust cognitive improvement by EPO to obtain the first clues of how EPO influences cognition, and how it may act on hippocampal neurons to modulate plasticity.

**Results:**

We show here that a 3-week treatment of young mice with EPO enhances long-term potentiation (LTP), a cellular correlate of learning processes in the CA1 region of the hippocampus. This treatment concomitantly alters short-term synaptic plasticity and synaptic transmission, shifting the balance of excitatory and inhibitory activity. These effects are accompanied by an improvement of hippocampus dependent memory, persisting for 3 weeks after termination of EPO injections, and are independent of changes in hematocrit. Networks of EPO-treated primary hippocampal neurons develop lower overall spiking activity but enhanced bursting in discrete neuronal assemblies. At the level of developing single neurons, EPO treatment reduces the typical increase in excitatory synaptic transmission without changing the number of synaptic boutons, consistent with prolonged functional silencing of synapses.

**Conclusion:**

We conclude that EPO improves hippocampus dependent memory by modulating plasticity, synaptic connectivity and activity of memory-related neuronal networks. These mechanisms of action of EPO have to be further exploited for treating neuropsychiatric diseases.

## Background

The hematopoietic growth factor erythropoietin (EPO) has long been observed to exert beneficial effects on cognition. Upon introduction of recombinant human EPO into the clinic, cognitive improvement of patients with chronic renal failure was noted during EPO treatment, but attributed to its hematopoietic effects (for review see [1-4]). Indeed, anemia after isovolemic hemodilution, induced in healthy volunteers, impairs cognitive performance, which is completely restored by subsequent autotransfusion [5].

However, the finding that EPO and its receptor (EPOR) are expressed in the brain [6,7] (for review see also [1,3,8-11]) led to the notion that EPO exerts direct, hematopoiesis-independent effects on the nervous system. The manufacturing of EPO analogues with no hematopoietic but potent neuroprotective properties, e.g. CEPO (carbamoylated EPO) [12], delivered proof-of-principle that brain effects of EPO are not necessarily mediated by its hematopoietic actions.

Beneficial effects of EPO on cognitive functioning have been shown in different animal models of neuropsychiatric diseases, e.g. on place navigation after global ischemia or neurotrauma [13-17]. In a recent double-blind, placebo-controlled, proof-of-concept study in chronic schizophrenic patients, we showed that EPO improved schizophrenia-relevant cognitive performance independently of its hematopoietic effects. In fact, EPO turned out to be the first compound to exert a selective and lasting beneficial effect on cognition in schizophrenia [18]. Similarly, an increase in cognitive performance upon EPO in patients with chronic progressive multiple sclerosis occurred independently of changes in hemoglobin levels, and persisted for months after termination of EPO treatment [4,19].

Recently, the application of a single high intravenous dose of EPO in healthy human volunteers was reported to enhance the functional MRI-detectable hippocampus response during memory retrieval 1 week later, before any effect on hemoglobin was measured [20]. However, data on hippocampus dependent memory in healthy human subjects upon EPO are still missing. Altogether, little is known about potential cognitive effects of EPO in healthy individuals. Hengemihle et al. [21] reported that 19 weeks of low-dose EPO treatment increased spatial memory performance, and a conditioned learning task, taste aversion, was enhanced by a single high-dose injection of EPO in healthy mice [22].

In summary, the currently available data clearly indicate that EPO can improve cognitive function of both rodents and man by directly acting on the nervous system. To be able to fully exploit this beneficial cognitive effect of EPO for treatment of neuropsychiatric diseases, it is essential to understand the cellular mechanisms of EPO action in healthy brain, where interference of disease-related effects can be excluded. Here, we systematically addressed this problem. We developed a reliable, robust model for improvement of cognition by EPO in healthy mice and examined correlated effects of EPO on hippocampal synaptic transmission and learning/memory-relevant synaptic plasticity. Further, we analysed effects of EPO on cultured hippocampal neurons at network and single cell levels. Our data indicate that EPO improves memory by modulating synaptic connectivity of memory-related neuronal networks within the hippocampus.

## Results

### EPO improves hippocampus dependent memory in healthy young mice

**Figure 1:**
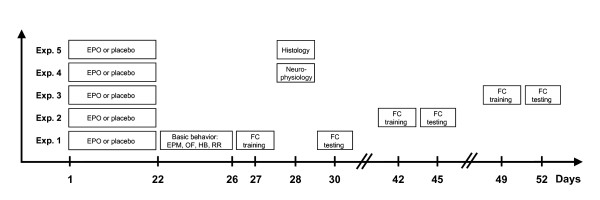
**Experimental design of the *in vivo *studies**. The time line of behavioral testing and brain dissection is presented. EPO or placebo was injected every other day for 3 weeks (11 injections in total). Tests performed were elevated plus maze (EPM), open field (OF), hole board (HB), rota-rod (RR), and fear conditioning (FC), including training and testing 72 h later.

**Figure 2:**
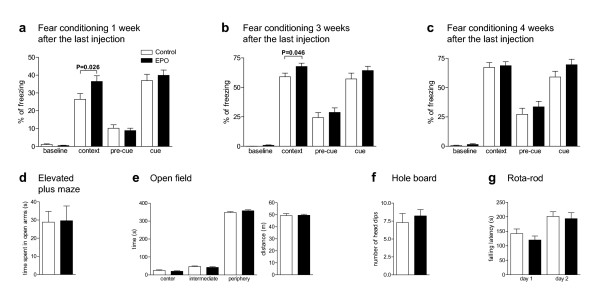
**Effects of EPO on hippocampus dependent memory**. Percentage of freezing as a readout of memory function in fear conditioning shows significant effects upon EPO treatment in the contextual memory (context) task at 1 week **(a) **and 3 weeks **(b)**, but no longer at 4 weeks **(c) **after the last EPO injection. Percentage of freezing measured during training (baseline), exposition to the new context (pre-cue), and testing for cued memory (cue) is not different between the groups. No differences are seen in EPM **(d)**, OF **(e)**, HB **(f)**, and RR **(g)**. Mean ± S.E.M. *N *= 28 for experiment in **(a) **and *N *= 14 for all other experiments **(b-g)**.

First goal of this study was to define an experimental condition to test potential abilities of EPO to improve cognitive functions. We used young (28 day old) male mice. In our experimental set-up with 11 intra-peritoneal EPO versus placebo injections (5000 IU/kg) every other day for 3 weeks (Figure 1), EPO-treated mice showed significant improvement of contextual memory in fear conditioning 1 week after the last injection, when tested 72 h after training in the same context (Figure 1, Exp. 1, Figure 2a). This effect was still measurable 3 weeks after cessation of EPO treatment but had disappeared after 4 weeks (Figure 1, Exp. 2 and Exp. 3; Figure 2b, c). In contrast, EPO had no effect on cued memory (Figure 2a–c; all *P *> 0.05). Whereas at 1 week after termination of treatment, hematocrit was still increased in EPO-treated mice (control mice: 36.5 ± 0.84%, *N *= 8; EPO mice: 53.3 ± 1.34%, *N *= 10; *P *< 0.0001), there was no difference anymore between groups at 3 weeks (control mice: 39.4 ± 1.19%, *N *= 14; EPO mice: 40.8 ± 0.92%, *N *= 13; *P *= 0.338), indicating that cognitive improvement and hematopoietic effects of EPO are not directly related.

In two additional experiments, EPO was given only three times either at the beginning or at the end of the 3-week treatment period while the respective other eight injections consisted of placebo. In this setting, no improvement in cognitive performance was obtained (data not shown), suggesting that a certain amount of EPO treatment is required for improving cognition.

The effect of EPO on hippocampus dependent (contextual) memory was selective. There was no EPO effect on anxiety, spontaneous activity, exploratory behavior, and motor performance (Figure 2d–g; all *P *> 0.05). Time spent in open arms of elevated plus maze (Figure 2d) and time spent in the three different zones of open field was similar in both groups (Figure 2e). Total distance traveled in open field did not differ between groups nor did exploratory activity in hole board test (Figure 2e, f). Over two days of rota-rod testing, both groups did not differ in falling latency (Figure 2g), indicating that motor performance and motor learning were comparable. Taken together, EPO treatment over 3 weeks leads to selective and long-lasting improvement of hippocampus dependent (but not of global) memory in healthy mice, independent of hematopoietic effects.

### Synaptic plasticity is significantly increased at Schaffer collateral CA1 synapses in EPO-treated mice

**Figure 3:**
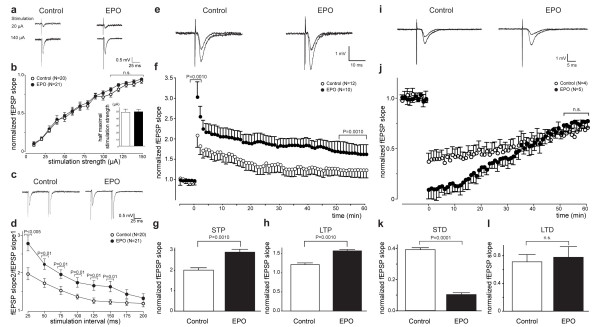
**Neurophysiology of acute hippocampal slices: *Extracellular recordings***. **(a-b) **Input-output relation is not altered at Schaffer collateral-CA1 synapses in EPO-treated mice. **(a) **Sample recordings at 50% of maximal response (average of four traces) are shown for control and EPO-treated mice. **(b) **Input-output curve as a measure of baseline excitatory synaptic transmission: fEPSP slope, plotted against the stimulation strength, is not altered in EPO-treated mice compared to control (*P *= 0.3094). Inset: Half maximal stimulation strengths are not significantly different. **(c-d) **Paired-pulse facilitation is enhanced in EPO-treated mice. **(c) **Sample traces are presented. **(d) **Paired-pulse ratio (fEPSP slope for the second stimulus/fEPSP slope for the first stimulus) at inter-stimulus intervals of 25–150 ms is significantly greater in EPO-treated mice. **(e-h) **Increased LTP at Schaffer collateral CA1 synapses in EPO-treated mice. **(e) **Sample traces of responses are shown before and after high frequency stimulation (HFS; 3 × 100 Hz for 1 s each, 20 s interval). **(f) **Long-term potentiation elicited by HFS: Slopes of fEPSP are normalized to baseline and plotted against time. Time-point 0 represents application of HFS. **(g) **Magnitude of STP, determined as maximal responses within 1 min after HFS, is significantly greater in EPO-treated mice. **(h) **Magnitude of LTP, determined as responses between 50 and 60 min after HFS, is significantly greater in EPO-treated mice. **(i-l) **Increased STD at Schaffer collateral-CA1 synapses in EPO-treated mice. **(i) **Sample traces of responses are shown before and after low frequency stimulation (LFS; 1 Hz for 900 stimulations). **(j) **Long-term depression elicited by LFS: Slopes of fEPSP are normalized to baseline and plotted against time. Time 0 represents application of LFS. **(k) **Magnitude of STD, determined as maximal responses within 1 min after LFS, is significantly greater in EPO-treated mice. **(l) **Magnitude of LTD, determined as responses between 50 and 60 min after LFS, is not significantly changed in EPO-treated mice (*P *= 0.0869).

One likely explanation for the selective improvement of contextual memory would be a direct influence of EPO on synaptic plasticity in the hippocampus. We therefore investigated the effect of EPO in acute hippocampal slices from mice at 1 week after the last injection (Figure 1, Exp. 4). We first performed extra-cellular recordings of field excitatory postsynaptic potentials (fEPSPs). Input-output curves were obtained by evoking responses from stratum radiatum of the CA1 region after stimulation of Schaffer collaterals with increasing stimulation strengths (Figure 3a, b). Average of fEPSP slopes (Figure 3b) between stimulus intensities of 110–150 μA from all slices yielded no difference between control and EPO groups. Half-maximal stimulation strength was also comparable (Figure 3b, inset). Thus, EPO treatment for 3 weeks, followed by a treatment-free week, does not alter basal excitability.

We then measured paired-pulse facilitation (PPF), the shortest form of plasticity at many synapses [31], at different inter-stimulus intervals (25–200 ms) in the Schaffer collateral CA1 pathway as ratio of the second fEPSP slope to the first fEPSP slope. Slices from EPO mice showed significantly enhanced paired-pulse facilitation at inter-stimulus intervals 25–150 ms (Figure 3c, d). Next, the effect of EPO on short-term potentiation (STP) and long-term potentiation (LTP) at the Schaffer collateral CA1 pathway was examined (Figure 3e–h). The magnitude of STP was defined as the maximal responses within the first minutes after induction by a train of 100 Hz stimuli. STP was significantly enhanced in slices of EPO mice compared to control (Figure 3f, g). Furthermore, the magnitude of LTP, determined as the average of responses between 50 and 60 min after induction by a train of 100 Hz stimuli, was also enhanced in slices of EPO mice compared to control (Figure 3f, h).

Another form of synaptic plasticity is long-term depression (LTD). We determined the effect of EPO treatment on short-term depression (STD) and LTD at Schaffer collateral CA1 pathway (Figure 3i–l). Magnitude of STD was defined as maximal responses within the first minutes after induction by a train of 900 stimuli (1 Hz). We found that STD was significantly enhanced in slices of EPO mice compared to control (Figure 3j, k). On the other hand, the magnitude of LTD, determined as average of responses between 50 and 60 min after induction by a train of 900 stimuli (1 Hz), was not significantly different in slices of EPO mice compared to control (Figure 3j, l). Collectively, these data show that EPO modulates synaptic plasticity and LTP in the hippocampus, but has no significant effect on LTD.

### EPO differentially influences inhibitory and excitatory synaptic transmission in the Schaffer collateral CA1 pathway

**Figure 4:**
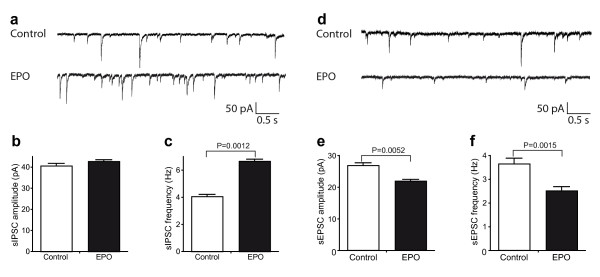
**Neurophysiology of acute hippocampal slices: *Intracellular recordings***. **(a-c) **EPO enhances inhibitory transmission. **(a) **Representative recordings of spontaneous, pharmacologically isolated inhibitory postsynaptic currents (sIPSCs) from CA1 neurons. **(b) **Averaged amplitude of sIPSCs is not significantly altered in EPO-treated mice (*N *= 6 neurons/5 mice) compared to control (*N *= 4 neurons/4 mice; *P *= 0.0869). **(c) **Averaged frequency of sIPSCs is significantly enhanced in EPO-treated mice (*N *= 6 neurons/5 mice) compared to control (*N *= 4 neurons/4 mice). **(d-f) **EPO decreases excitatory transmission. **(d) **Representative recordings of spontaneous, pharmacologically isolated excitatory postsynaptic currents (sEPSCs) from CA1 neurons. **(e) **Averaged amplitude of sEPSCs is significantly decreased in EPO-treated mice (*N *= 4 neurons/4 mice) compared to control (*N *= 4 neurons/3 mice). **(f) **averaged frequency of sEPSCs is significantly decreased in EPO-treated mice (*N *= 4 neurons/4 mice) compared to control (*N *= 4 neurons/3 mice).

To study cellular mechanisms of EPO action, we performed whole-cell patch-clamp recordings on CA1 pyramidal neurons in acute hippocampal slices from mice at 1 week after the last injection (Figure 1, Exp. 4; Figure 4a–f). Compared to control mice, the frequency of spontaneous inhibitory postsynaptic currents (sIPSCs) in CA1 pyramidal neurons of EPO mice was increased, while the amplitude of sIPSCs was unchanged (Figure 4b, c). In contrast, EPO led to a significant decrease of both amplitude and frequency of spontaneous excitatory postsynaptic currents (sEPSCs) in CA1 pyramidal neurons (Figure 4e, f). Importantly, there were no significant differences in input resistance or basic noise level between neurons of control and EPO mice (data not shown). Thus, EPO modulates inhibitory and excitatory synaptic transmission inversely.

We wondered whether the neurophysiological changes found in hippocampal slices upon EPO treatment would be due to alterations in total volume or synapse counts in the involved areas, CA1 and CA3. Neither volume of CA1 (control: 3.97 ± 0.11 mm^3^, *N *= 9; EPO: 4.02 ± 0.16 mm^3^, *N *= 10; *P *= 0.81) nor CA3 (control: 3.29 ± 0.21 mm^3^, *N *= 8; EPO: 3.56 ± 0.25 mm^3^, *N *= 10; *P *= 0.42), nor total hippocampal volume (control: 9.54 ± 0.34 mm^3^, *N *= 8, versus EPO: 9.74 ± 0.39 mm^3^, *N *= 10; *P *= 0.71) were significantly different. Moreover, density of synaptic boutons in CA1 (control: 1.28 ± 0.08 boutons/μm^2^, *N *= 7; EPO: 1.32 ± 0.11 boutons/μm^2^, *N *= 9; *P *= 0.75) and CA3 (control: 0.71 ± 0.13 boutons/μm^2^, *N *= 7; EPO: 0.78 ± 0.08 boutons/μm^2^, *N *= 9; *P *= 0.61) was not changed. Quantitative RT PCR and/or Western blotting using extracts of whole hippocampus did not reveal differences in expression of synaptic proteins (synapsin1, synaptophysin), postsynaptic receptor proteins (GABA_A_1,2,3,4; NMDAR1, R2A, R2B) or BDNF, as potential mediating neurotrophic factor [20,32] (data not shown).

### EPO modulates spontaneous electrical network activity in primary hippocampal neurons as determined by multi-electrode measurements

Above data demonstrated distinct and long-lasting effects of temporary high-dose EPO treatment on hippocampus dependent memory and synaptic plasticity in hippocampal slice preparations. As the peritoneal applications of EPO might have caused indirect effects on nerve cells, we next studied primary hippocampal cultures. We tested whether chronic EPO treatment, extending from an advanced developmental stage (day 5 in culture) to over 3 weeks leads to alterations in spontaneous neuronal network activity, and whether such changes would persist upon cessation of EPO treatment.

**Figure 5:**
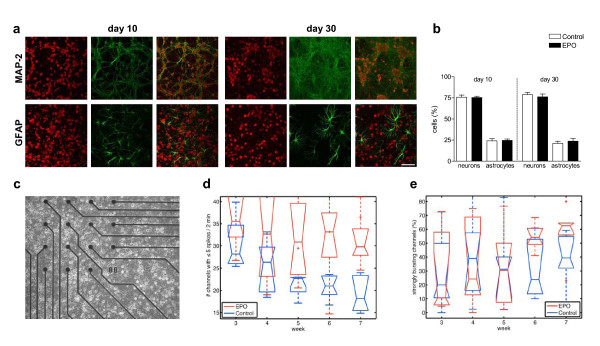
**Multi-electrode array studies of primary hippocampal neurons**. **(a-b) **Characterization of the cultures. **(a) **Immunocytochemical staining demonstrates maturation of cellular networks from day 10 to day 30. Propidium iodide staining of all nuclei (red), visualization of cell types by MAP-2 (mature neurons) or GFAP (astrocytes) staining (green), as well as merged pictures are presented (scale bar = 100 μm). **(b) **Cellular composition of networks remains stable over time and is not altered by EPO treatment (0.3 IU/ml every other day) from day 5 through 25 in culture (Mean ± S.E.M. of *N *= 3 independent cultures per time point). **(c) **Demonstration of primary hippocampal neurons grown on multi-electrode array dishes, containing 60 electrodes/dish. **(d-e) **Spontaneous electrical activity of primary hippocampal neuronal networks in culture is measured daily from week 3 through week 7. Group statistics of the multi-electrode array recordings over each week show significant dissociation over time of EPO versus control cultures. **(d) **Silencing group statistics reveal a global decrease of channels with low activity in control cultures that cannot be observed in EPO-treated cultures. **(e) **Bursting group statistics show that the percentage of strongly bursting channels increases in the EPO group after termination of treatment. Medians ± S.E.M. presented of *N *= 7 independent cultures. *P *values are given in the text.

First, our long-term cultures were characterized regarding morphological appearance (Figure 5a), total cell numbers (day 10: control: 202.4 ± 11.03, *N *= 6; EPO: 191.0 ± 8.834, *N *= 6; *P *= 0.436; day 30: control: 147.9 ± 26.26, *N *= 6; EPO: 152.8 ± 27.87, *N *= 6; *P *= 0.902), and relative contribution of different cell types (Figure 5b). In none of these parameters were differences upon EPO found at days 10 or 30 in culture. Also, quantitative RT PCR and protein expression, determined by Western blotting, failed to uncover differences in synapsin1 or synaptophysin gene expression at any of the time points tested (days 8, 14, and 30). Thus, EPO treatment did not cause changes in morphology under our culture conditions.

Figure 5c illustrates primary hippocampal neurons grown on multi-electrode array (MEA) dishes. Group statistics for spontaneous electrical activity in the MEAs are presented in Figures 5d and 5e, contrasting silencing (number of channels with < 5 spikes per 2 min) and bursting behavior (percentage of strongly bursting channels of all active channels, with strongly bursting channels defined as channels with a coefficient of variation > 2.6). With increasing age and maturation of culture, the number of silent channels decreased in control MEAs, as expected (Figure 5d). This was not the case in EPO cultures. Whereas during the treatment phase itself, cultures behaved largely similarly (weeks 3 and 4 with *P *= 0.41 and *P *= 0.18, respectively), differences became obvious at later time points (weeks 5 through 7 with *P *= 0.047, *P *= 0.0043 and *P *= 0.0043, respectively). This indicates that temporary EPO treatment causes a significant number of channels to remain silent for an extended period after cessation of EPO addition to cultures.

The bursting channel analysis, presented in Figure 5e, showed that EPO provoked a consistently higher number of bursts in hippocampal cultures, obvious only at late time points, i.e. 2–3 weeks after termination of EPO treatment. This effect (expressed as percentage of all active channels in order to exclude the influence of silencing) was less pronounced as compared to the silencing effect of EPO. Whereas medians at week 6 were not yet significantly different (*P *= 0.10), difference reached significance at week 7 (*P *= 0.019). Together, the trend of weeks 6 and 7, when compared with the almost equal-bursting situation at week 5 (*P *= 0.70), confirms that bursting tends to increase as a late consequence of transient EPO treatment, in parallel with the persistently high percentage of silent channels.

### Reduction of synaptic vesicle priming and transmitter release in the EPO pre-treated neurons

The finding of long-lasting EPO-induced dampening of spontaneous electrical activity in our primary hippocampal cultures together with a selective increase in bursting activity following EPO treatment prompted us to test individual neurons. We examined the effect of EPO in hippocampal autaptic cultures [30], to directly assess the EPO effect on presynaptic transmitter vesicle exocytosis and postsynaptic receptor responses. Autaptic neurons are neurons forming synapses on themselves, making electrophysiological stimulation and respective effect determination (recording) simple. Cultures were treated with EPO (0.3 IU/ml = 10^-10^M) or the respective buffer solution only once at day 7 and then measured from days 9 to 14. There were no morphological differences detectable upon treatment, and sizes of somata as estimated by measurement of whole cell capacitance were comparable between EPO-treated and control neurons (control neurons: 49.61 ± 2.75pF, *N *= 54; EPO neurons: 46.0 ± 2.73pF, *N *= 49; *P *= 0.355).

**Figure 6:**
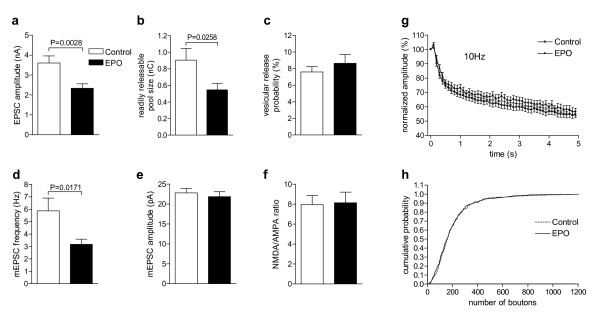
**Autaptic hippocampal neuronal cultures**. **(a-g) **Whole-cell electrophysiological recordings from single hippocampal neurons treated with either EPO (0.3 IU/ml) or control (diluent only) on day 7 and measured from day 9–14. Results indicate a reduction in the amount of primed vesicles without altering efficiency of vesicle fusion and vesicle dynamics. Mean ± S.E.M. presented. *N *= 40–60. **(h) **Analysing the number of synaptic boutons upon immunocytochemical staining for synapsin1 revealed an almost identical increase of boutons over time in EPO-treated and control neurons. Performed at 40x. Cumulative distribution over days 9–14. *N *= 100–120.

Evoked excitatory postsynaptic current (EPSC) amplitudes in EPO-treated neurons were reduced to about 60% of control (Figure 6a), confirming the data obtained in acute slices (Figure 4e). This EPSC reduction was due to a parallel reduction in pool size of fusion-competent and primed (readily releasable) vesicles, whose release can be triggered by hypertonic solution containing 0.5 M sucrose [33]. EPO neurons showed a drastic reduction in readily releasable pool size to 60% of control (Figure 6b). Vesicular release probability, calculated by dividing the charge transfer during a single EPSC by the charge transfer measured during readily releasable pool release, was not different between control and EPO neurons (*P *= 0.4116; Figure 6c). To test whether the reduction of neurotransmitter release in EPO neurons can be attributed to a reduction in quantal size, we analysed miniature EPSCs (mEPSC). mEPSC frequency in EPO neurons was reduced to about 50% of control, without changes in mEPSC amplitudes (*P *= 0.5817; Figure 6d, e). The lack of a difference in NMDA/AMPA ratio indicates a comparable maturation state of cultures (Figure 6f). Using trains of action potentials we estimated the efficiency of Ca^2+ ^triggered release. In general, vesicular release probability closely correlates with depression and steady-state level of EPSC amplitude sizes during high frequency stimulation. We therefore monitored EPSC amplitudes during 50 consecutive action potentials applied at a frequency of 10 Hz. EPO and control neurons showed regular moderate depression of EPSC amplitudes (control: about 38%, *N *= 60; EPO: depression at the end of train about 35%, *N *= 60, Figure 6g). Stability of EPSC amplitudes during short-term plasticity, which is due to the quantitative balance between priming of synaptic vesicles and number of vesicles released, was identical in presynaptic terminals of each group.

Thus, EPO treatment of autaptic neurons leads to a reduction in the amount of primed vesicles or number of synapses without altering efficiency of vesicle fusion and vesicle dynamics. Counting of synaptic boutons per neuron revealed a considerable increase from day 9 to day 14 in culture, which, however, was not changed by EPO (Figure 6h). Therefore, EPO is likely to reduce the number of active synapses without altering total synapse number.

## Discussion

We show that young mice, systemically treated with EPO for 3 weeks, exhibit improved hippocampus-associated memory. This selective improvement was maintained for an EPO treatment-free period of another 3 weeks, and was unrelated to increases in hematocrit, indicating a hematopoiesis-independent effect of EPO on neuroplasticity. The long-lasting effect of EPO on neuroplasticity was confirmed by analyses of paired-pulse facilitation, STP, LTP and STD, as well as of spontaneous synaptic activity in acute hippocampal slices, obtained from EPO-treated mice at the time point of EPO-induced enhancement of memory. MEA recordings of neuronal assemblies *in vitro *and the analysis of individual autaptic hippocampal neurons did not only confirm direct effects of EPO on neural cells, but also reveal potential mechanisms of action: EPO leads to a reduction in the amount of primed vesicles without altering number of synapses or efficiency of vesicle fusion and vesicle dynamics. Thus, most likely via increasing the proportion of silent synapses, EPO reduces overall spiking activity of neurons and enhances bursting efficiency of selected neuronal networks. Most of these data are consistent with EPO shifting the balance between excitatory and inhibitory transmission (i.e. functionally silencing a subset of excitatory presynaptic sites and increasing activity of inhibitory neurons), although other mechanisms cannot be entirely excluded at this point.

In humans, improvement of cognitive function upon treatment with EPO has only been demonstrated in disease states [18,19,34], i.e. in conditions of reduced/disturbed baseline performance. Exploring healthy individuals has therefore been a risky endeavour, although, if successful, promised to deliver a cleaner picture of mechanisms of EPO action, lacking interference with potential disease variables. Similar to what is observed with endurance and muscular performance during doping [35], where healthy individuals show dramatic improvement, we found significant memory effects in healthy mice. EPO-treated compared to placebo-treated mice had a significantly longer duration of freezing, as readout of memory function, during a contextual memory test that is known to be critically dependent on the hippocampus [36,37]. This finding implies that in healthy individuals the potential cognitive capacity is not fully exhausted. Although results were obtained in mice, the work of Miskowiak and colleagues [20] may indicate that respective effects can be expected in healthy humans.

Similar to the findings of Miskowiak et al. [20], the effect of EPO on hippocampal functions was measurable at 1 week after injection. In our setting, treatment for 3 weeks (11 injections) was necessary to obtain positive results on cognitive performance. Reduced to only three injections, no measurable effect on the behavioral readout of hippocampal functions was obtained. Healthy humans showed increased hippocampal response (perfusion equivalent) in functional magnetic resonance imaging upon memory retrieval already 1 week after a single EPO dose. However, effects on memory function were also not detectable after this single dose [20]. In other words, for cognitive improvement (and not only for increase in perfusion), more than a single injection is needed also in humans. In both studies, the hematocrit seems irrelevant. In the human study, a single dose of EPO had not changed the hematocrit after 1 week [20]. In our study, the hematocrit was already back to control levels when we still observed a significant effect on cognition, and direct effects of EPO on synaptic plasticity were found in hippocampal cultures.

The persistent effect of EPO on cognition, lasting for over 3 weeks after cessation of treatment, indicates alterations in neuroplasticity induced by EPO that do not require its continuous presence. Interestingly, our studies in MS patients showed beneficial effects of EPO on motor function, which lasted for up to 6 months after termination of a 6-months treatment [19]. In search for a mechanism explaining the lasting influence of EPO on hippocampus-associated memory, we detected pronounced EPO effects on short-term and long-term plasticity, as well as on excitatory and inhibitory synaptic transmission in the Schaffer collateral CA1 pathway. These electrophysiological parameters of plasticity have been associated with learning and memory [38-40].

Further exploring mechanisms of action of EPO, we employed multi-electrode arrays to study network activity in primary hippocampal cultures. We found that chronic application of EPO in a fashion similar to our *in vivo *approach resulted in persistence of a large population of silent channels but enhanced bursting efficiency of discrete neuronal circuits. In acute hippocampal slices as well as autaptic hippocampal cultures, excitatory synaptic transmission was decreased upon EPO treatment, whereas inhibitory synaptic transmission was increased. In line with these data, EPO-mediated inhibition of glutamate release has been reported for cerebellar granule cells [41].

Together, these findings may point to an enhanced lateral inhibition within the hippocampal neuronal network by EPO, leading to amplification of active synaptic connections. A concurrent suppression of surrounding synapses by EPO, consistent with lasting functional silencing, may ultimately achieve segregation/refinement of neuronal networks (for review see [42]). Interestingly, signal transduction pathways known to be activated in hippocampal neurons by EPO, include PI3K-PKB/Akt1 and RAS-MAPK [43,44]. Both, the MAPK-mediated pathway [45-47] and PI3K have been linked to LTP [48,49].

## Conclusion

Although not providing complete mechanistic insight at this point, our data indicate that the selective enhancing effect of EPO on hippocampus dependent memory is mediated via profound changes in neuroplasticity. These plastic changes, in turn, may be based on a more efficient bursting activity of selected synapses together with persistent silencing of other synapses.

## Methods

### Animals

All experiments were approved by and conducted in accordance with the regulations of the local Animal Care and Use Committee. For all experiments, young (28 days old) C57/Bl6 male mice were used. They were housed in groups of five in standard plastic cages and maintained in a temperature-controlled environment (21 ± 2°C) on a 12 h light/dark cycle with food and water available ad libitum.

### Drug treatment

For experiments 1–5, mice were injected intra-peritoneal with EPO (Epoetin-alpha, Janssen-Cilag, Neuss, Germany, 5 IU/g in 0.01 ml) or placebo (diluent for EPO, 0.01 ml/g) every other day for 3 weeks (11 injections in total). Two additional groups of mice received only three injections of EPO or placebo either at the beginning or at the end of the 3-week treatment period. The remaining eight injections were all placebo. Before each injection, the body weight was measured. The experimenter, who administered the injections and performed the tests, was blinded concerning group assignment.

### Experimental design of mouse studies

The experimental design including behavioral tests, neurophysiology, and brain tissue analyses is presented in Figure 1.

#### Experiment 1

EPO effects on basic behavior and cognition of young healthy mice after termination of EPO treatment were assessed. Mice were tested, starting on the day after the last injection, for anxiety (EPM, elevated plus maze), spontaneous loco-motor activity (OF, open field), exploratory activity (HB, hole board), motor functioning (RR, rota-rod) and memory (FC, fear conditioning).

#### Experiments 2 and 3

In these experiments, mice were tested in FC either 3 or 4 weeks after the last EPO injection to explore the duration of EPO effects on cognition. Hematocrit was determined immediately after FC.

#### Experiments 4 and 5

These experiments were set up to obtain brain tissue of mice for neurophysiology and histology at the time point with the most prominent effect of EPO on hippocampus dependent memory.

### Behavioral testing

Group size in all behavioral experiments amounted to *N *= 15–28. Exact numbers of individual experiments are given in the legend of Figure 2.

#### Elevated plus maze

The mouse was placed in the central platform, facing an open arm of the plus-maze. Behavior was recorded by an overhead video camera and a PC equipped with 'Viewer' software (Biobserve, Bonn, Germany) to calculate the time each animal spends in open or closed arms. The time spent in open arms was used for estimation of open arm aversion (fear equivalent).

#### Open field

Spontaneous activity in open field was tested in a grey Perspex arena (120 cm in diameter, 25 cm high). The mouse was placed in the center and allowed to explore the open field for 7 min. The behavior was recorded by a PC-linked overhead video camera. 'Viewer' software was used to calculate velocity, distance traveled and time spent in central, intermediate or peripheral zones of the open field.

#### Hole board

The hole board test measures exploratory activity. The apparatus consisted of a 21 cm × 21 cm × 36 cm transparent Perspex chamber with a non-transparent floor raised 5 cm above the bottom of the chamber with 12 equally spaced holes, 2 cm in diameter. Mice were allowed to explore the chamber for 3 min and the number of explored holes (head dips) was scored by a trained experimenter.

#### Rota-rod

Rota-rod is a test for motor function, balance and coordination and comprises a rotating drum (Ugo Basile, Comerio, Varese, Italy), which is accelerated from 4 to 40 revolutions per minute over the course of 5 min. Each mouse was placed individually on a drum and the latency of falling from the drum was recorded using a stop-watch. To assess motor learning, the rota-rod test was repeated 24 h later.

#### Cued and contextual fear conditioning

The fear conditioning test was performed as described in detail earlier [23]. Briefly, mice were trained within the same session for both contextual and cued fear conditioning. Training consisted of exposing mice for 120 s to the context to assess the baseline level of activity. This period was followed by a 10 s, 5 kHz, 85 dB tone (conditioned stimulus, CS). Immediately after the tone, a 2 s, 0.4 mA foot shock (unconditioned stimulus, US) was applied. This CS-US pairing was repeated 13 s later. All mice remained in the conditioning chambers for an additional 23 s following the second CS-US pairing. The contextual memory test was performed 72 h after this training. Mice were monitored over 2 min for freezing in the same context as used for training. The cued memory test was performed 76 h after training in a new chamber. First, mice were monitored for freezing over a 2 min pre-cue period with no tone to assess freezing in the new context. Next, a 2 min cue period followed during which the tone was presented. Duration of freezing behavior, defined as the absolute lack of movement (excluding respiratory movements), was recorded by a video camera and a PC equipped with 'Video Freeze' software (MED Associates, St. Albans, Vermont, USA).

### Brain dissection and sections preparation

For RNA and protein analysis, mice were deeply anaesthetized and decapitated. Hippocampi were taken out, immediately frozen on dry ice and stored at -80°C. For histology, mice were perfused under deep anesthesia with 4% paraformaldehyde. Brains were dissected, postfixed overnight at 4°C and transferred into 30% sucrose/PBS solution. After having sunk, they were frozen in liquid nitrogen and stored at -80°C. Whole mouse brains were cut into 30 μm thick coronal sections on a cryostat (Leica, Wetzlar, Germany) and kept in a storage solution (25% ethyleneglycol and 25% glycerol in PBS). Every 10th section throughout the dorsal part of the hippocampal formation was selected for staining, yielding five to six sections per brain, used for either volumetrical analysis or confocal microscopy.

### Volumetric measurements using histological sections

The sections were mounted on Super Frost microscopic slides, washed in phosphate buffer, then immersed for 25 min in a dilute cresyl violet stain (0.01%) in acetate buffer (pH 4.5), dehydrated in serial dilutions of ethyl alcohol and finally coverslipped using DePeX (Serva, Heidelberg, Germany). Calculation of the volume of CA1, CA3 subregions and the total hippocampus was based on thickness of the sections and areas obtained by tracing contours around the regions of interest, using a light microscope (Olympus BX50) modified for stereology with a 10× objective, a computer-driven motorized stage, *Z*-axis position encoder (microcator), and a microfire video camera interfaced to a PC with the software Stereo Investigator 6.55 (MicroBrightfield, Inc., Williston, VT, USA). Volumetric determinations were performed on both sides of the hippocampus.

### Confocal analysis

For counting of synaptic boutons, sections were washed in PBS, permeabilized and blocked in 5% blocking serum for 1 h at 4°C, and incubated at 4°C overnight with rabbit polyclonal synapsin1 antibody (1:4000; Synaptic Systems, Goettingen, Germany). After PBS washes, the sections were incubated with anti-rabbit AlexaFluor555-labeled secondary antibody (1:2000; Invitrogen, Karlsruhe, Germany). Following PBS washes, sections were mounted on Super Frost microscopic slides, air dried and coverslipped with fluorescence mounting medium (Vector, Burlingame, CA, USA) containing DAPI. Synapsin1 immunoreactive presynaptic boutons were analysed within stratum radiatum of area CA1 and stratum lucidum of area CA3 of hippocampus. Images were obtained at a zoom factor 4 using an inverted confocal laser scanning microscope (LSM 510; Zeiss, Goettingen, Germany) with a 63× oil-immersion objective. For intensity comparisons, gain and offset were held constant across images. Synapsin1 immunoreactive punctae were quantified using ImageJ software (Rasband, W.S., ImageJ, U. S. National Institutes of Health, Bethesda, Maryland, USA). Images were manually thresholded and particle analysis plugin was used to calculate the number of immunoreactive punctae.

### Hippocampal slice preparation and solution

Acute hippocampal slices were prepared from 56 days old mice (Figure 1, Exp. 4). As in all experiments performed here, the experimenter was blinded regarding group assignment. Mice were deeply anesthetized with diethyl ether before decapitation. The brain was quickly removed and immersed for 2–3 min in ice-cold artificial cerebrospinal fluid (ACSF). The ACSF had the following composition (in mM): 130 NaCl, 3.5 KCl, 1 CaCl_2_, 1.2 MgSO_4_, 24 NaHCO_3_, 1.25 NaH_2_PO_4_, 10 Glucose, with the pH adjusted to 7.4. Transverse slices of 400 μm thickness were cut with a vibroslicer (752 M, Campden Instruments, Loughborough, UK). The slices were then transferred to an interface recording chamber of the Oslo type and allowed to recover for at least 90 min. The recording chamber was continuously perfused with ACSF, aerated with 95% O_2 _and 5% CO_2 _(3–4 ml/min). The temperature was kept at 34°C.

### Extracellular recordings of hippocampal slices

The recording electrodes were pulled from thin-walled borosilicate glass capillaries (GC150TF-10, Harvard Apparatus, Holliston, MA, USA) using a horizontal Flaming-Brown micropipette puller (P-80/PC, Sutter Instrument Co., Novato, CA, USA). They were filled with ACSF. Monopolar stimulation electrodes made from bare stainless steel microwire (50 μm diameter, AM-Systems) were used for stimulation. The stimuli were generated by photoelectric stimulus isolation units (Grass PSIU6) triggered by a stimulator (Grass S88). Extracellular field potential recordings were done using a custom-built DC amplifier. Data were digitized by a DigiData 1322A (Molecular Devices, Sunnyvale, CA, USA). Initial analysis of the data was done in Clampfit 9.0 (Molecular Devices, Sunnyvale, CA, USA). To evoke field excitatory postsynaptic potentials (fEPSPs), the stimulation electrode was placed in stratum radiatum at CA3/CA1 junction for the activation of Schaffer collaterals. The recording electrode was placed in the stratum radiatum of the CA1 region. The magnitude of fEPSPs was measured as amplitude (baseline to peak) and slope (20–80% level of the falling phase). For input-output relationship, fEPSPs were evoked with 0.1 ms stimuli at 0.25 Hz and an average of four consecutive responses was taken. fEPSP amplitudes and slopes were plotted against the stimulus intensity (10 to 150 μA). Paired-pulse facilitation (PPF) was measured at different interstimulus intervals (25, 50, 75, 100, 125, 150, 175 and 200 ms) as the ratio of the second fEPSP to the first fEPSP. Here also the paired stimuli were given at 0.25 Hz and an average of four consecutive responses was taken. To study long-term potentiation (LTP), baseline responses were evoked every 20 s for 5 min and LTP was induced by three trains separated by 20 s, each train consisting of 100 Hz stimulation for 1s. The post-train responses were then measured every 20 s for 60 min. The magnitude of LTP was measured as the average of responses between 50 and 60 min after induction. To study long-term depression (LTD), baseline responses were evoked every 15 s for 5 min and LTD was induced by 900 stimuli delivered at 1 Hz. The post-train responses were then measured every 15 s for 60 min. The magnitude of LTD was measured as the average of responses between 50 and 60 min after induction.

### Whole-cell patch clamp-recordings

Acute transverse 300 μm hippocampal slices were prepared as described above. After preparation, slices were incubated for 30 min at 34°C, followed by room temperature incubation for more than 1 h. All recordings were performed in CA1 hippocampal pyramidal neurons. The extracellular solution in all experiments was the same as the one used in LTP experiments. The pipette solution for all experiments contained (in mM): 140 KCl, 1 CaCl_2_, 10 EGTA, 2 MgCl_2_, 4 Na_3_ATP, 0.5 Na_3_GTP, 10 HEPES at pH 7.3. Spontaneous inhibitory PSCs were recorded at a Cl-reversal potential of about 0 mV in 10 μM CNQX and 40 μM AP5. Spontaneous excitatory PSCs were recorded in the presence of 1 μM strychnine and 1 μM bicuculline. Signals with amplitudes of at least two times above the background noise were selected. Patches with a serial resistance of > 10 MΩ, a membrane resistance of < 0.2 GΩ, or leak currents of > 200 pA were excluded. The membrane currents were filtered by a four-pole Bessel filter at a corner frequency of 2 kHz, and digitized at a sampling rate of 5 kHz using the DigiData 1322A interface (Molecular Devices, Sunnyvale, CA). Data acquisition and analysis were done using commercially available software: pClamp 9.0 (Molecular Devices, Sunnyvale, CA), MiniAnalysis (SynaptoSoft, Decatur, GA) and Prism 4 (GraphPad Software, San Diego, CA).

### Primary hippocampal neuronal culture

Mice at embryonic day 17 (E17) were used for preparation of hippocampal primary neuronal cell cultures [24,25] Briefly, after complete removal of meninges, hippocampi were dissected in warm HBSS solution (Invitrogen, Karlsruhe, Germany), supplemented with penicillin and streptomycin, and trypsinized. After mechanical trituration with fire polished Pasteur pipettes, cells were plated on poly-D-lysine- and laminin-coated 6-well plates (for Western blotting and quantitative RT PCR) or on poly-D-lysine- and laminin-coated MEA dishes (for multi-electrode array, MEA) or on poly-D-lysine- and laminin-coated glass cover slips in 6-well plates (for immunocytochemistry) at a density of 200000 cells per well. Neurons were cultured in MEM/B27 medium (Invitrogen, Karlsruhe, Germany) supplemented with sodium bicarbonate, sodium pyruvate, L-glutamine, penicillin, streptomycin and 0.6% glucose. Cultures were incubated at 37°C under 7.5% CO_2_/92.5% air and 90% humidity. One-third of medium volume was exchanged every 5th day. Contamination with glial fibrillary acidic protein positive astrocytes on day 5 in culture was consistently less than 7%. For all MEA experiments, EPO or control treatment (0.3 IU/ml = 10^-10 ^M) was started on day 5 and continued by addition of EPO every other day until day 25. Cell cultures were maintained until day 50 for MEA, until day 8, 14, or 30 for Western blotting and quantitative RT PCR, until day 10 and 30 for immunocytochemistry.

### Immunostaining of cultured cells

After 10 or 30 days in culture, cells were washed in PBS, fixed with 4% paraformaldehyde in PBS, permeabilized and blocked in 0.2% Triton X-100/PBS with 10% blocking serum, and incubated at 4°C overnight with mouse monoclonal MAP-2 (1:500; Chemicon, Hampshire, UK) or mouse monoclonal GFAP (1:500; Novocastra, Newcastle Upon Tyne, UK) antibodies diluted in 1% blocking serum/PBS. After PBS washes, the cells were incubated with Cy2-labeled secondary antibody (1:250; Jackson ImmunoResearch, Newmarket, UK), washed in PBS, air dried and coverslipped with fluorescence mounting medium (Vector, Burlingame, CA, USA) containing propidium iodide.

### Multi-electrode array recordings and analysis

For determination of spontaneous electrical network activity in primary mouse hippocampal neuronal cultures, we used multi-electrode arrays (MEA) of 60 titanium nitride electrodes with 30 μm diameter each and 200 μm inter-electrode distance (Multi Channel Systems, Germany). Raw data from the MEA electrodes were amplified by MEA 1060 filter amplifiers (bandwidth 3 Hz-10 kHz; gain × 1100). Sampling frequency amounted to 25 kHz. The experiments were performed at 37°C, using a TC01 temperature controller. Recording of spontaneous network activity was carried out daily in the morning for 2 min, starting on day 14 and ending on day 50. This gave us five weeks of daily recordings, from week 3 until week 7 (total of 37 days). The choice of morning hours for measurements did not affect the statistics, as confirmed by an additional evening experiment showing little daily differences. Seven independent "sister" cultures (i.e. cultures derived from the same brain preparation), treated with EPO or control were analysed. Spike extraction from the continuous data is commonly achieved by spike sorting [26,27]. Having to process 481 2 min recordings, manual interaction, often used to improve sorting behavior, was not feasible. Thus, automated spike extraction using MEATools, a MATLAB-based toolbox for comprehensive analysis of multi-neuronal data  was employed. For each channel, principal components were calculated, and spikes were identified via thresholds in the principal component contributions. In order to identify multivariate features explaining potential modifications by EPO in the cell cultures, single sample analyses were performed first (see Additional file 1). Due to a relatively high background noise and a low overall number of spikes in the channels, standard statistics, such as spike rates and spike time interval distributions, did not capture significant differences in EPO versus control cultures (see Additional files 2 and 3). A direct quantification of the variations in bursting and silent channels was therefore necessary. Similar clustering effects have been previously studied in oscillator networks on a theoretical level [28,29]. Here, two indices were calculated: (1) In order to measure silencing in the groups, we determined the number of channels *c*_*i*_(*t*) of dish *i *at time *t *with basically no spikes (less than five spikes per 2 min). We then took the mean of *c*_*i*_(*t*) over each week and compared the time evolution of this mean channel activity using a Wilcoxon rank sum test in each week. The test was performed on the samples after outlier removal, where an outlier was defined as a sample not lying within 1.5 times the interquartile range from the median. (2) In addition to silencing effects, we analysed bursting behavior. For this, we calculated the coefficient of variation (CV) of the spike-time interval distribution in each channel, i.e. the ratio of standard deviation and mean. This measure of dispersion is larger than 1 for hyper-exponential distributions and lower than 1 for lower-variance distributions. In the case of bursting channels, over-proportionally many small spike-time intervals were observed, so the spike-time intervals obeyed a hyper-exponential distribution, which could be identified by high CV-values of the corresponding channels. We defined bursting behavior if the CV-value was above 1, and strongly bursting behavior if it was above a threshold of 2.6 (see also Additional file 1). In order to quantify bursting over all channels, we counted the percentage *b*_*i*_(*t*) of strongly bursting channels of all active channels of dish *i *at time *t*. By calculating relative bursting with respect to active channels, we were able to study bursting independent of the number of silent channels. Again, we took the mean over each week, and tested for differing medians of the EPO and the control group using a rank sum test.

### Autaptic neuron experiments

#### Cell culture

Microislands of astrocyte feeder cells were prepared two days before plating hippocampal neurons [30]. Islands of substrate (10 mM acetic acid, 0.1 mg/ml poly-D-lysine, and 0.2 mg/ml collagen) were applied to agarose-coated glass coverslips using a stamp containing regularly spaced squares (200 μm × 200 μm). To obtain astrocytes and hippocampal neurons, P0 mice were decapitated, and brains were removed and cleaned of meninges and vascular tissue. To obtain hippocampal neurons, hippocampi were removed in HBSS, digested in papain (25 IU/ml, Worthington Biomedical) in DMEM (supplemented with 1 mM CaCl_2_, 0.5 mM EDTA, and 1.65 mM L-cysteine) for 45 min at 37°C, incubated for 15 min at 37°C in serum-free medium (Neurobasal medium A supplemented with 2.5 mg/ml Albumin and 2.5 mg/ml Trypsin inhibitor) and dissociated. To obtain astrocytes, the cortices of separate animals were removed in HBSS, similarly dissociated (digested for 15 min at 37°C in Trypsin/EDTA) and plated at a density of 2500 per cm^2 ^in DMEM containing 10% fetal calf serum, penicillin/streptomycin, and MITO (Becton Dickinson). Before plating the dissociated hippocampal neurons at a density of 300 per cm^2^, the medium of the astrocyte feeder cells was replaced with Neurobasal medium A (supplemented with B27, Glutamax-I and penicillin/streptomycin). Neurons were allowed to mature until days 9, 11, or 14 to be used for electrophysiology or immunocytochemistry. Only islands containing single neurons were examined. EPO versus control (diluent solution) treatment was performed on day 7. If not otherwise indicated, cell culture reagents were obtained from GIBCO/Invitrogen.

#### Immunostaining

For estimating the number of synaptic boutons in autaptic neurons, cells were washed in PBS, fixed with 4% paraformaldehyde in PBS, permeabilized and blocked in 0.2% Triton X-100/PBS with 10% blocking serum, and incubated at 4°C overnight with mouse monoclonal synapsin1 antibody (1:1000 SynapticSystems, Goettingen, Germany) diluted in 1% blocking serum/PBS. After PBS washes, cells were incubated with Cy3-labeled secondary antibody (1:1000; Jackson ImmunoResearch, Newmarket, UK), washed in PBS and incubated at 4°C overnight with mouse monoclonal MAP-2 (1:500; Chemicon, Hampshire, USA) antibody. Following PBS washes, the cells were incubated with Cy2-labeled secondary antibody (1:250; Jackson ImmunoResearch, Newmarket, UK), washed in PBS, air dried and coverslipped with fluorescence mounting medium (Vector, Burlingame, CA, USA) containing DAPI. Images of individual neurons were captured using an upright epifluorescence Olympus BX61 microscope (Hamburg, Germany) with a 40× oil-immersion objective. Images were photomerged to reconstruct individual neurons using Adobe Photoshop CS3 software. The number of synapsin1 immunoreactive punctae of 18–20 neurons per coverslip (six coverslips per condition) were quantified using ImageJ software with manual thresholding and particle analysis plugin. Estimation of the percentage of excitatory and inhibitory neurons was performed by visual distinction between the degree of arborization, thickness of processes and shape of soma. Amount of inhibitory neurons among the total neuronal population was found to be 10–20% per culture.

#### Electrophysiology

Cells were whole-cell voltage clamped at -70 mV with pClamp10 amplifier. All analyses were performed using Axograph 4.9 (Molecular Devices, Sunnyvale, CA, USA). The size of the readily releasable pool (RRP) of synaptic vesicles was determined by a 6 s application of the external saline solution made hypertonic by the addition of 0.5 M sucrose. Recordings of mEPSCs were performed in the presence of 300 nM tetrodotoxin (TTX). EPSCs were evoked by depolarizing the cell from -70 to 0 mV for 2 ms. The effect of high-frequency stimulation on the amplitude of EPSCs was measured by applying depolarisations at a frequency of 10 Hz for 50 stimuli. To measure NMDA/AMPA ratio, EPSCs were stimulated in the presence of 10 mM glycine, 2.5 mM Ca^2+ ^(no Mg^2+^) to activate the synaptic NMDA receptors in hippocampal autaptic culture. The evoked EPSCs had a fast AMPA component followed by a slow NMDA component. To examine the changes in synaptic NMDA/AMPA ratios in presence and absence of EPO, the NMDA components relative to the AMPA component were measured.

Patch-pipette solutions contained (mM): 146 potassium gluconate, 18 HEPES, 1 EGTA, 4.6 MgCl_2_, 4 NaATP, 0.3 Na_2_GTP, 15 creatine phosphate and 5 U/ml phosphocreatine kinase (315–320 mOsmol/l, pH 7.3). The extracellular saline solution contained (mM): 140 NaCl, 2.4 KCl, 10 HEPES, 10 glucose, 4 CaCl_2 _and 4 MgCl_2 _(320 mOsmol/l, pH 7.3). All chemicals, except for TTX (Tocris Cookson) and calcimycin (Calbiochem) were purchased from Sigma. All solutions were applied using a fast-flow system (Warner Instruments, Hamden, CT, USA) with custom made flow pipes.

### Protein extraction and immunoblot analysis

Tissue samples or cells were lysed with lysis buffer [50 mM Tris HCL (pH 8.3), 150 mM NaCl, 40 mM NaF, 5 mM EDTA, 5 mM EGTA, 1 mM Na_3_VO_4_, 1% Igepal, 0.1% Natriumdesoxycholat, 0.1% SDS] containing 1 mM Phenylmethysulfonylfluoride, 10 μg/ml Aprotinin and 1 mg/ml Leupeptin. The lysates were freeze-thawed four times and homogenized by pulling through a 1 ml syringe 10 times, transferred into microcentrifuge tubes and centrifuged (1200 rpm) at 4°C for 45 min. The supernatant was mixed with three volumes of Laemmli buffer [250 mM Tris HCL (pH 8.3), 8% SDS, 40% glycerol, 20% 2-mercaptoethanol, 0.04% pyronin Y], boiled for 5 min at 95°C and frozen at -20°C until blotting. The protein samples were run on NuPAGE 4–12% Bis-Tris Gel (Invitrogen, Karlsruhe, Germany) for 35 min at 200 V and transferred to a nitrocellulose membrane. The blots were blocked with 2% ECL Advance blocking agent (Amersham, Freiburg, Germany) in Tween 20-Tris-buffered saline (TTBS) at room temperature for 1 h and incubated at 4°C overnight with primary antibody for synapsin1 (1:10000; Synaptic Systems, Goettingen, Germany) or synaptophysin (1:500; Sigma, Germany) or α-tubulin as an internal control (1:5000; Sigma, Germany). Immunoreactive bands were visualized by using secondary antibodies coupled to horseradish peroxidase by enhanced chemoluminescence (Amersham, Freiburg, Germany). Densitometric analysis of the protein bands was performed by using ImageJ software.

### RNA isolation and expression analysis by quantitative real-time RT-PCR

RNA was isolated from tissue samples or cells by using the RNeasyPlus kit (Qiagen, Hilden, Germany). First strand cDNA was generated from total RNA using N9 random and Oligo(dT)18 primers. The relative concentrations of mRNAs of interest in different cDNA samples were measured out of four replicates using the threshold cycle method (Ct) for each dilution and were normalized to levels of murine 18S RNA. Reactions were performed using SYBR green PCR master mix (ABgene, Foster City, CA, USA) according to the protocol of the manufacturer. Cycling was done for 2 min at 50°C, followed by denaturation at 95°C for 10 min. The amplification was carried out by 45 cycles of 95°C for 15 s and 60°C for 60 s. The specificity of each primer pair was controlled with a melting curve analysis. Quantitative RT-PCR was performed with primers listed below:

NM_007540.3 *Mus musculus brain derived neurotrophic factor (BDNF)*, mRNA

mouse *BDNF *fwd: GCA TCT GTT GGG GAG ACA AG

mouse *BDNF *rev: TGG TCA TCA CTC TTC TCA CCT G

NM_010149.2 *Mus musculus erythropoietin receptor (EPOR)*, mRNA

mouse *EPOR *fwd: CCT CAT CTC GTT GTT GCT GA

mouse *EPOR *rev: CAG GCC AGA TCT TCT GCT G

NM_009305.1 *Mus musculus synaptophysin (Syp)*, mRNA

mouse *synaptophysin *fwd: CAA GGC TAC GGC CAA CAG

mouse *synaptophysin *rev: GGT CTT CGT GGG CTT CAC T

NM_013680.3 *Mus musculus synapsin1 (Syn1)*, mRNA

mouse *synapsin1 *fwd: GGA AGG GAT CAC ATT ATT GAG G

mouse *synapsin1 *rev: TGC TTG TCT TCA TCC TGG TG

### Statistical analysis

Statistical significance was evaluated using two-tailed unpaired Student's *t*-test, with or without Welch's correction, depending on the distribution of the data (tested with a Kolmogorov-Smirnov test). Significance level was set to *P *< 0.05. Numerical values are represented as mean ± S.E.M. in Figures and text. Plotting of the data as well as statistical analyses were done in Prism 4 (GraphPad Software, San Diego, CA, USA) and MATLAB 7 (The MathWorks, Natick, MA, USA).

## Authors' contributions

BA carried out the behavioral experiments. DS performed the immunohistochemical analysis and synapse counting. BA and DS participated in writing the manuscript. AS carried out most of the electrophysiological analysis of slice cultures. CG and JSR were involved in preparation and electrophysiology of autaptic cultures. FT and FW performed statistical analysis of MEA cultures. LM, MZ, MM and LS were involved in electrophysiological experiments with slice cultures. IH performed immunohistochemistry. KH was involved in cell culture experiments and western blot analysis. SS carried out mouse brain preparations for immunohistochemistry. KR, AEK and AR were involved in behavioral experiments. NB participated in the design of the study and helped to draft the manuscript. WZ supervised electrophysiology of slices. HE supervised the whole project, designed the study and wrote the manuscript. All authors read and approved the final manuscript.

## Supplementary Material

Additional file 1 Analysis of single-sample MEA recordings. Click here for file

Additional file 2 Mean conditional firing rates for EPO and control samples. Click here for file

Additional file 3 Spike-rate (1/s) for EPO-treated and control dishes. Click here for file
