# Supplementary material for: Erythropoietin enhances hippocampal long-term potentiation and memory
Source: BMC Biol. 2008 Sep 9;6:37. doi: 10.1186/1741-7007-6-37 (PMC2562991; doi:10.1186/1741-7007-6-37)
Supplement: Additional file 1 — Analysis of single-sample MEA recordings. [file 1741-7007-6-37-S1.pdf]

# Additional file 1

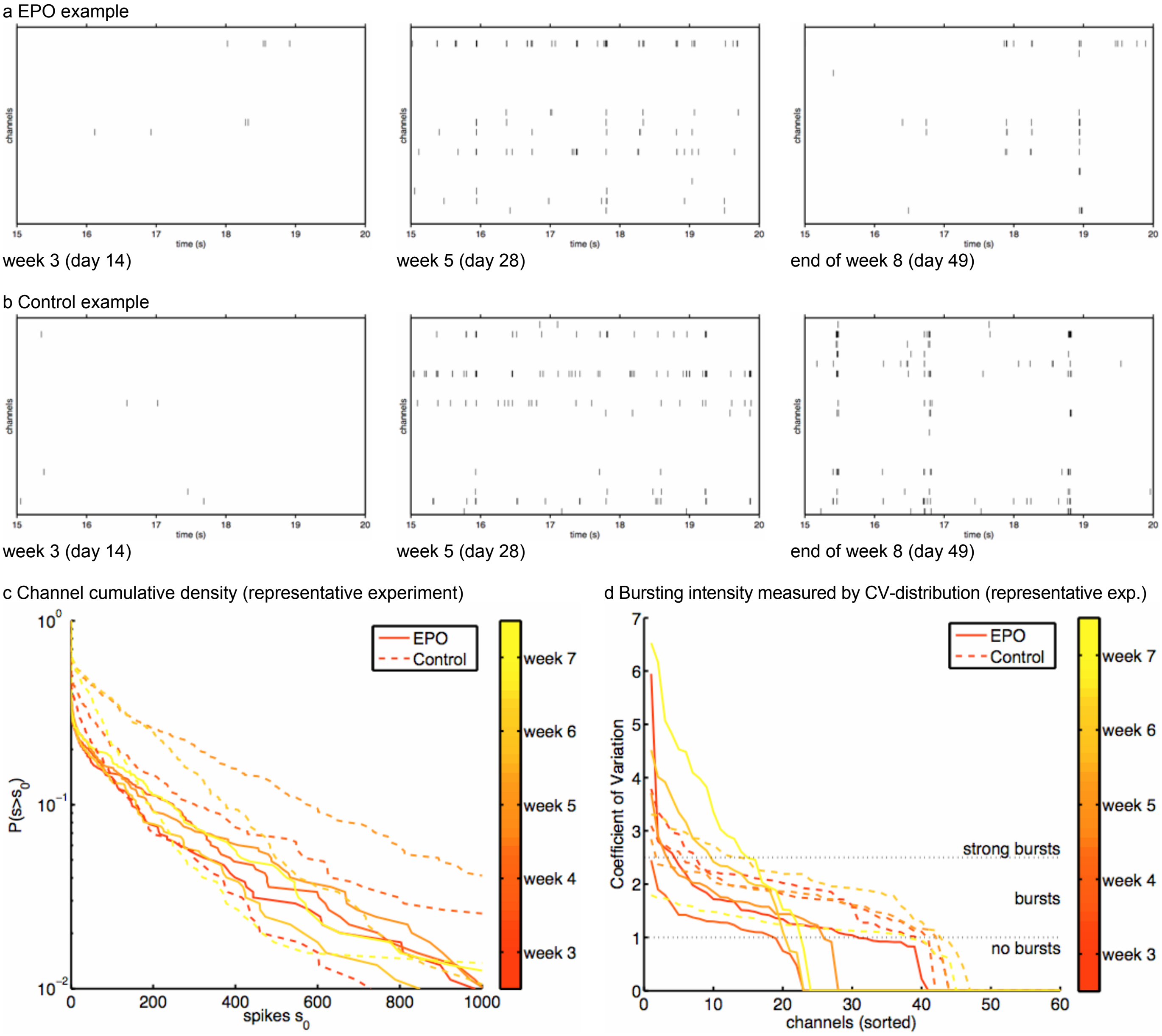


Single-sample MEA recordings at different days are analysed. (a) shows an EPO sample at the beginning of treatment (week 3), directly after treatment (week 5) and at the end of the recording (week 8). Only the central 16 channels out of the total 60 channels are shown (electrodes are positioned on an 8x8 grid except for the 4 vertices, see Figure 4c). When compared with the control (b), channel activity is lower, with at the same time higher bursting behavior. This is illustrated for the same two samples in (c), where the channel cumulative density at the different 5 weeks is shown for both EPO and control; the lower onset of EPO can be numerically captured by counting the number of silent channels (compare Fig 4d). Similarly, considering bursting behavior in each channel, accumulated over each week, shows that EPO leads to more strongly bursting channels than the control at later weeks (d), which is confirmed by the group statistics (compare Fig 4e).
