# Supplementary material for: Erythropoietin enhances hippocampal long-term potentiation and memory
Source: BMC Biol. 2008 Sep 9;6:37. doi: 10.1186/1741-7007-6-37 (PMC2562991; doi:10.1186/1741-7007-6-37)
Supplement: Additional file 2 — Mean conditional firing rates for EPO and control samples. [file 1741-7007-6-37-S2.pdf]

Additional file 2

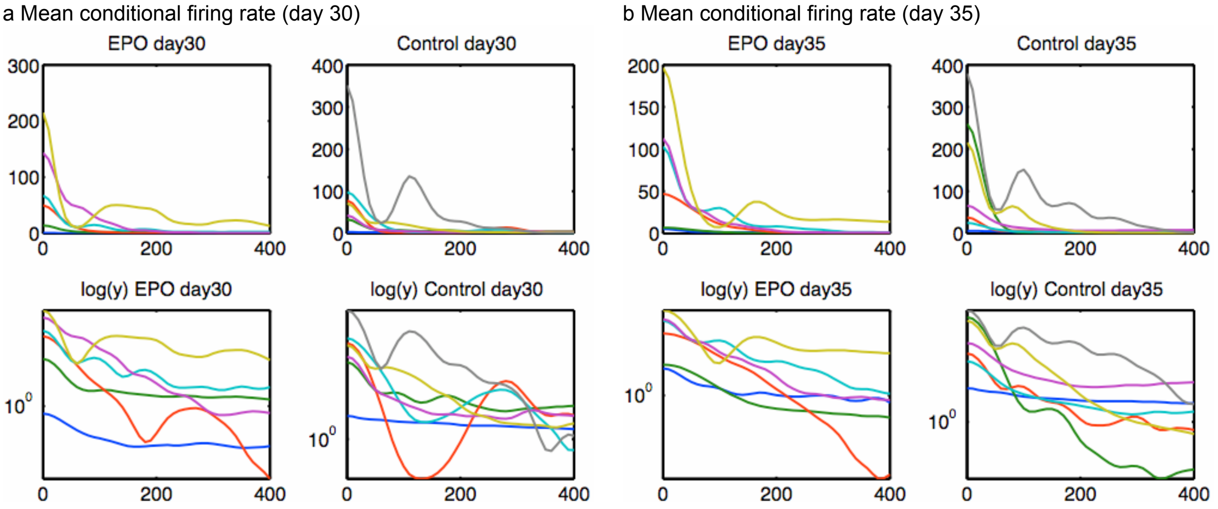

Mean conditional firing rates between two different channels *i* and *j* after taking the mean over *i* and *j* at different lags 0 to 400ms; calculation was performed using a Gaussian kernel at each spike with standard deviation 10ms. We show the firing rates for the different samples (indicated by different colors) for both EPO and control at day 30 (a) and at day 35 (b). Using a logarithmic y-axis (lower subfigures), a tendency towards "bumpier" behavior in case of the control might indicate longer-range autocorrelations of the control versus EPO, however significant differences and time-shifts could not be identified.
