# Supplementary material for: Erythropoietin enhances hippocampal long-term potentiation and memory
Source: BMC Biol. 2008 Sep 9;6:37. doi: 10.1186/1741-7007-6-37 (PMC2562991; doi:10.1186/1741-7007-6-37)
Supplement: Additional file 3 — Spike-rate (1/s) for EPO-treated and control dishes. [file 1741-7007-6-37-S3.pdf]

Additional file 3

Spike-rate (1/s)

	week 3	week 4	week 5	week 6	week 7
EPO	42.5 $\pm$ 43.7	58.5 $\pm$ 49.1	65.3 $\pm$ 38.7	50.2 $\pm$ 17.5	49.9 $\pm$ 20.5
Control	43.3 $\pm$ 33.5	73.1 $\pm$ 19.8	83.8 $\pm$ 43.7	92.1 $\pm$ 44.2	81.9 $\pm$ 54.8

Spike-rate (1/s) for EPO-treated and control dishes when compared over a whole week. No significant differences were found.
